# *Helicobacter pylori *infection and circulating ghrelin levels - A systematic review

**DOI:** 10.1186/1471-230X-11-7

**Published:** 2011-01-26

**Authors:** Chidi V Nweneka, Andrew M Prentice

**Affiliations:** 1 Nutrition Programme, Medical Research Council Laboratories, The Gambia, P.O. Box 273, Banjul, The Gambia; 2 MRC International Nutrition Group, London School of Hygiene & Tropical Medicine, Keppel Street, London, UK

## Abstract

**Background:**

The nature of the association between ghrelin, an orexigenic hormone produced mainly in the stomach, and *Helicobacter pylori (H pylori)*, a bacterium that colonises the stomach, is still controversial. We examined available evidence to determine whether an association exists between the two; and if one exists, in what direction.

**Methods:**

We reviewed original English language studies on humans reporting circulating ghrelin levels in *H pylori *infected and un-infected participants; and circulating ghrelin levels before and after *H pylori *eradication. Meta-analyses were conducted for eligible studies by combining study specific estimates using the inverse variance method with weighted average for continuous outcomes in a random effects model.

**Results:**

Seventeen out of 27 papers that reported ghrelin levels in *H pylori *positive and negative subjects found lower circulating ghrelin levels in *H pylori *positive subjects; while 10 found no difference. A meta-analysis of 19 studies with a total of 1801 participants showed a significantly higher circulating ghrelin concentration in *H pylori *negative participants than in *H pylori *positive participants (Effect estimate (95%CI) = -0.48 (-0.60, -0.36)). However, eradicating *H pylori *did not have any significant effect on circulating ghrelin levels (Effect estimate (95% CI) = 0.08 (-0.33, 0.16); Test for overall effect: Z = 0.67 (P = 0.5)).

**Conclusions:**

We conclude that circulating ghrelin levels are lower in *H pylori *infected people compared to those not infected; but the relationship between circulating ghrelin and eradication of *H pylori *is more complex.

## Background

The relationship between ghrelin, a 28-amino acid peptide secreted primarily by the oxyntic cells of the stomach [1] and involved in body mass regulation, and *Helicobacter pylori *(*H pylori*), a bacterium that colonises the stomach, has remained controversial. The first report suggesting an association between the two was that by Nwokolo et al [2] who examined the effect of *H pylori *eradication on plasma ghrelin levels in 12 healthy adult male and female subjects. They reported that eradicating *H pylori *from the subjects was associated with an increase in plasma ghrelin levels. At about the same time, Gockel et al [3] reported that *H pylori *had no effect on plasma ghrelin levels in a study of 39 age- and BMI-matched *H pylori *positive and negative women. Subsequently a number of other papers, including animal studies, have explored this relationship [4-7]. A number of review articles have also appeared exploring this relationship [8,9]; but none of these has been conducted systematically.

Considering the putative role of ghrelin in body mass regulation, understanding this association could help in maximizing its benefits, and also provide further insight into the physiology of appetite and body mass regulation. The objective of this review is to examine available evidence to determine whether or not a relationship exists between ghrelin and *H pylori *infection; and where one exist, to investigate the direction of the association. Specifically, this review sets out to answer three questions: 1) Is *H pylori *infection associated with circulating ghrelin levels? 2) what is the effect of eradicating *H pylori *infection on circulating ghrelin levels?; and 3) what is the effect of *H pylori *infection on ghrelin producing cells in the stomach?

## Methods

### Literature search strategy and data extraction

A comprehensive search of the scientific literature (Medline (OVID), OvidMedline (R) 1950 - October Week 2 plus In-process & Non-indexed citations, Embase (1980 to 2010 week 41), and ISI Web of Knowledge) was conducted using the search terms "ghrelin AND *helicobacter pylori*". The search was repeated several times. The last search was conducted on October 29, 2010. Further searches were conducted using Google scholar; while the bibliography of original and review articles were searched for studies with ghrelin and *helicobacter pylori *in their titles. Duplicate searches were first removed; thereafter, the abstracts of retrieved articles were reviewed for relevance prior to accessing the full paper. Only English-language primary studies on humans were included provided that the authors assessed at least one of the following: 1) compared circulating ghrelin concentration in *H pylori *positive and negative subjects; 2) compared the effect of eradicating *H pylori *on circulating ghrelin levels; or 3) compared gastric ghrelin in *H pylori *positive and negative subjects or changes in gastric ghrelin after *H pylori *eradication. Letters in response to published articles, commentaries, and editorials were excluded. Conference abstracts that had not been published as full papers were included where the full abstracts could be retrieved, provided that such conference abstract contained enough information for either the qualitative or the quantitative synthesis. However, where a conference abstract has been published as a full paper, the full paper was retrieved and the conference proceeding excluded.

Efforts were made to contact authors of conference abstracts whose full paper publications could not be traced to inquire if the paper had been published as a full paper and if not, to get further details about the study. Efforts were also made to contact authors of papers where some relevant information was missing to request for the missing information or for further clarifications. We contacted 18 authors [2,6,10-25]; but only nine authors [2,14,18,21-26] responded and provided the needed information. We were unable to contact one author [27].

We also attempted to group the papers reviewed by study teams in other to assess the spread of the papers reviewed. In deciding whether different papers were published by the same research groups, similarity in authorship was examined. Where at least one author contributed to different publications, those publications were deemed to have emanated from the same research group.

### Outcomes evaluated

Outcomes evaluated included: differences in circulating ghrelin levels between *H pylori *positive and negative subjects; changes in circulating ghrelin concentration after *H pylori *eradication; differences in ghrelin mRNA between *H pylori *positive and negative subjects; changes in ghrelin mRNA after *H pylori *eradication; differences in ghrelin immunoreactive cells between *H pylori *positive and negative subjects; changes in ghrelin immunoreactive cells after cure; correlation between ghrelin immunoreactive cells with severity of *H pylori *infection; correlation between gastric and plasma ghrelin; correlation between ghrelin mRNA and plasma ghrelin; and correlation between cells and plasma ghrelin.

### Data synthesis

The data extracted were classified into three classes: 1) data comparing circulating ghrelin concentration in *H pylori *positive and negative subjects; 2) data comparing the circulating ghrelin concentration before and after *H pylori *eradication; and 3) data assessing any of the gastric ghrelin parameters. Each of these classes was supposed to answer a specific question (Table 1). The variables were re-coded in forms that would make analysis easier (Table 2). Table 3 lists the papers excluded from the review and the reasons for their exclusion.

**Table 1:** Research questions explored by the review

Research question	Explanatory data
Is *H pylori *infection associated with circulating ghrelin levels?	Data comparing circulating ghrelin concentration in *H pylori *positive and negative subjects
What is the effect of eradicating *H pylori *on circulating ghrelin levels?	Data comparing the circulating ghrelin concentration before and after *H pylori *eradication
What is the effect of *H pylori *infection on ghrelin expression in the stomach?	Data assessing any of the gastric ghrelin parameters

**Table 2:** Dictionary of variables used in the review

Variable	Coding scheme
**Study Team**	A, B, C etc
**Design**	Cohort, Cross-sectional, Case control, Experimental
**Healthy**	Healthy only, Sick only, Both
**Region**	Asia, Africa, Europe, North America, South America
**Gender**	Male only, Female only, Both
**Age**	Children only, Adults only, Both
**Number of methods used to assess *H pylori***	One method; two or more methods
**Type of ghrelin assay used**	Commercial RIA, in-house RIA, Commercial ELISA, Commercial EIA
**Sample storage**	-70°C and below; above -70°C
**Sample type**	Serum; Plasma
**Weight**	Normal, Low, Various, High
**Difference in circulating ghrelin concentration between *H pylori *+ & - subjects**	Lower, no difference, higher
**Changes in circulating ghrelin after cure**	Increased, decreased, no change
**Duration of follow-up**	4 weeks & below; Above 4 weeks
**Sample size**	Two categorizations were done: 1) 50 & below, 51-200, 201 and above; 2)Above 20, Below 20

### Statistical analysis

Simple proportions were used to determine the frequency of occurrence of each categorical variable considered; and association between different variables assessed using Fisher's exact chi^2 ^test. Continuous variables like sample size and duration of follow-up was initially summarized using medians and inter-quartile ranges and later categorized as shown in Table 2. Multiple logistic regression analysis was used to assess confounding. The descriptive analysis was conducted using Stata version 8 (StataCorp LP, College Station, Texas, USA).

### Meta-analysis

We conducted meta-analyses of summary statistics from individual studies that compared circulating ghrelin levels between *H pylori *positive and *H pylori *negative subjects; and for studies that compared circulating ghrelin levels before and after eradication of *H pylori*. For each study, the mean circulating ghrelin concentrations and standard deviations (sd) for the different comparison groups were used to generate standardized mean differences (SMDs) and 95% confidence intervals (95% CI) since different studies used different units to measure ghrelin concentration, and some studies measured plasma ghrelin while others measured serum ghrelin. For studies that reported standard errors of mean, standard deviation was derived using the formula provided in the Cochrane Handbook of clinical reviews: SD = SEM × √N (http://www3.interscience.wiley.com/homepages/106568753/handbook.pdf). Studies that reported only medians and 95% CI or interquartile ranges (IQR) [2,6] were not included in the meta-analysis. Table 4 lists studies excluded from the meta-analysis and the reasons for their exclusion.

Study specific estimates were combined using the inverse variance method with weighted average for continuous outcomes in a random effects model. Heterogeneity was assessed using the chi-squared method and the I^2 ^method described by Higgins et al [28,29]. For studies that reported different sub-groups separately [30-32] those sub-groups were included as separate papers in the meta-analysis. Chuang et al, [30] reported ghrelin levels for males and females separately. While the data for males did not increase the heterogeneity of the studies, inclusion of the data for females introduced significant heterogeneity to the analysis. All the papers that resulted in significant heterogeneity of the studies in the meta-analysis were removed from the meta-analysis. A separate meta-analysis conducted for this sub-group of excluded studies revealed a significant heterogeneity (P=0.0001; I^2 ^= 86%) and the effect size was very small and not significant (SMD -0.13 [-0.33, 0.06], Test for overall effect: Z = 1.36 (P = 0.17)). These papers were therefore completely excluded from the meta-analysis and described in a narrative. Sensitivity analysis was conducted to assess the contribution of each study to the pooled estimate by excluding individual studies one at a time and recalculating the pooled SMD estimates for the remaining studies. Funnel plots were used initially to assess publication bias and later confirmed using Begg's and Egger's tests. The meta-analyses were conducted using Review Manager Version 5 (RevMan 5; http://www.cc-ims.net/revman/download), while the Begg's and Egger's tests were conducted using Stata version 8 after downloading the installation files for the tests from the internet.

## Results

### Search results

**Figure 1:**
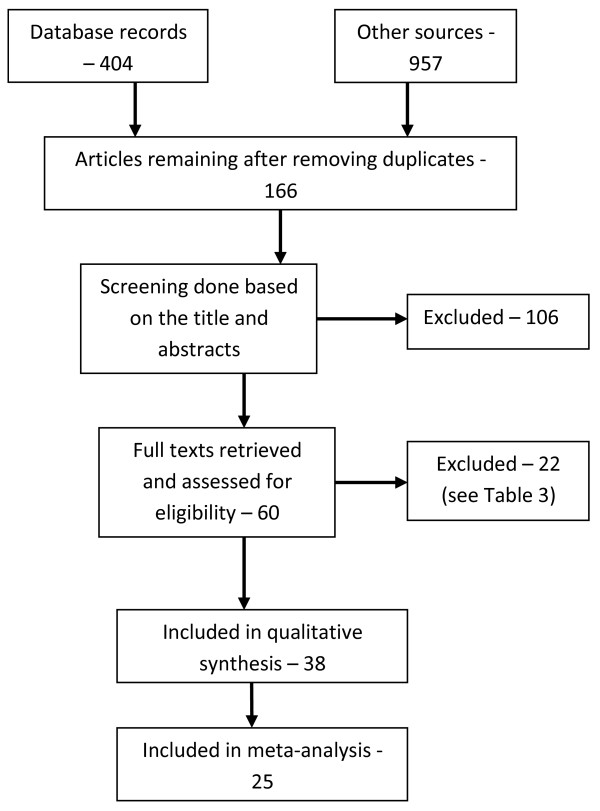
PRISMA flow chart showing information flow in the selection of papers for the review

The literature search yielded 1361 papers (404 from databases and 957 from Google Scholar) plus one unpublished paper. After removing duplicate articles, reviews, commentaries and letters (written in response to published articles), 166 papers (including one conference abstract and one unpublished paper) were left. These were further screened using titles and abstract to assess for eligibility, resulting in the exclusion of 106 articles (Figure 1). The full texts of the remaining 60 articles (excluding the conference abstract) were retrieved. Twenty-two papers were subsequently dropped from the review (Table 3), and another 11 from the meta-analysis (Table 4).

**Table 3:** Papers excluded from the review

	Reason for exclusion
Suzuki et al, 2006 [54]	Did not report ghrelin levels in *H pylori *positive &*H pylori *negative patients
Nunes et al, 2006 [27]	Not sufficient information in abstract and authors could not be contacted for further clarifications
Campana et al, 2007 [56]	All subjects were *H pylori *negative
Isomoto et al, 2005 [52]	Compared only *H pylori *strains
Checchi et al, 2007 [10]	Did not compare serum ghrelin levels in *H pylori *positive &*H pylori *negative subjects
Wu et al, 2005[57]	Did not assess ghrelin
Shinomiya et al, 2005[58]	Did not compare the difference in ghrelin between *H pylori *positive &*H pylori *negative subjects
Suzuki et al, 2005[59]	Did not assess ghrelin
Sundbom et al, 2007[60]	Did not assess *H pylori*
Huang et al, 2007[61]	Did not assess *H pylori*
Nishizawa et al, 2006[62]	Did not assess *H pylori*
Ando et al, 2006[63]	Did not assess ghrelin
Kempa et al, 2007[64]	All subjects were *H pylori *negative
Ates et al, 2008[65]	Did not assess *H pylori*
Wang et al, 2006[66]	Did not assess ghrelin
Doki et al, 2006[67]	Did not assess *H pylori*
Gao et al, 2008[68]	Excluded people with *H pylori*
Cherian et al, 2009[69]	Did not assess ghrelin
Kebapcilar et al, 2009[70]	Did not assess ghrelin
Dutta et al, 2009[71]	Did not assess ghrelin
Gen et al, 2010[72]	Did not assess ghrelin
Taniaka-Shintani et al, 2005[1]	Examined only ghrelin immunoreactive cells

**Table 4:** Papers excluded from the meta-analysis

Nwokolo et al, 2003[2]	Reported median values & 95% CI; also measured integrated ghrelin levels rather than the discrete measurement that other authors used. The authors stated that the median values were used because the sample size was small and the data were skewed
Mendez-Sanchez et al, 2007 [49]	Studied ghrelin immuno-reactive cells, did not provide data on plasma ghrelin levels
Salles et al, 2006[19]	Ghrelin values not provided. Only P-values
Gao et al, 2009[15]	Ghrelin values not provided
Choe et al, 2007[6]	Median & IQR provided. Further clarifications not provided by the authors
Czesnikiewicz-Guzik et al, 2005[12]	Insufficient information for meta-analysis
Czesnikiewicz-Guzik et al, 2007 [13]	Insufficient information for meta-analysis
Masaoka et al [51]	This was a case study with only one subject
Stec-Michalska et al, 2009[24]	Measured only gastric ghrelin
Liew et al, 2006[47]	Assessed only gastric ghrelin
Konturek et al, 2006[17]	The numbers of H pylori positive and negative participants were not provided and the authors did not respond to requests for further information.

### Is H pylori infection associated with circulating ghrelin levels?

Table 5 is an evidence table of studies that compared circulating ghrelin levels in *H pylori *positive and *H pylori *negative individuals. Twenty-six studies compared circulating ghrelin levels between *H pylori *positive and negative subjects. The analysis of one of the studies [30] was stratified by gender, yielding different results; this study was therefore entered into the evidence table as two separate papers with each gender representing one paper, bringing the total number of papers reviewed to 27. One paper [22] was not included in the evidence table because the grouping of the subjects studied did not permit a straight forward comparison of the *H pylori *positive and *H pylori *negative subjects. The paper was therefore reviewed in a separate narrative. Table 6 summarises the characteristics of the studies included in the evidence table. Most of the studies (77%) investigated both males and females, 85% studied only adults and 52% studied sick subjects mainly subjects with gastrointestinal symptoms (64.7%) and Cancer (17.65%). Thirteen (48.2%) of the studies [15,21,30,33-41] were conducted in Asia; 11 (40.7%) [3,11,12,17,19,26,31,32,42-44] in Europe and three (11.1%) [14,18,45] in North America. Twenty-two percent of the studies reviewed were contributed by one research group alone, while Japan contributed 30% of the papers. More than two-thirds of the studies used radioimmunoassay to measure serum or plasma ghrelin, 55.6% assessed *H pylori *using two or more methods, and in all the studies except one, samples were collected after overnight fast. All comparisons were based on the pre-meal ghrelin levels. In most of the studies (70.4%), the samples were stored at a temperature of -70°C or below until analysed; ghrelin was measure in plasma samples in 63% of the studies, while the participants were of normal weight in 58% of the studies. The sample sizes for the different studies ranged from 13 to 538 (median: 89; IQR: 62, 180).

**Table 5:** Evidence table of studies that compared circulating ghrelin levels in Hp+ and Hp- individuals

Reference & Country	Study Team	Design	Healthy	Gender	Age	Method of *H pylori *assessment	Ghrelin assay kit	Overnight fast	Sample storage	Sample type	Weight	Sample size	Ghrelin levels in Hp+ vs Hp-
Kawashima et al, 2009; Japan [38]	A	Cohort	Both	Both	Adults	Serology	Commercial EIA	Yes	-80	Plasma	Normal	220	Lower
Plonka et al, 2006; Poland [32]	B	Cohort	Healthy	Both	Both	Modified UBT plus ELISA	Commercial RIA	Yes	-80	Serum		538	Lower
Isomoto et al, 2005; Japan[36]	C	Cohort	Sick	Both	Adults	RUT, histology (Giemsa stain)	In-house RIA	Yes	-80	Plasma	Normal	81	Lower
Roper et al, 2008; USA [18]	D	Cross-sectional	Healthy	Men	Adults	serology, histology & RUT or positive culture	Commercial EIA	Yes	-20	Serum	Normal	256	No Difference
Pacifico et al, 2008; Italy [26]	E	Cohort	Both	Both	Children	Culture of gastric specimen or histology + RUT	Commercial RIA	Yes	-70	Serum	Normal	85	No Difference
Gokcel, 2003; Turkey [3]	F	Case control	Sick	Women	Adults	Not stated	Commercial EIA	Yes	Not stated	Plasma	Normal	39	No Difference
Plonka et al, 2006; Poland [43]	B	Case control	Healthy	Both	Children	Serology and UBT	Commercial RIA	Yes	-80	Serum	Various	287	Lower
Shiotani et al, 2005; Japan [21]	G	Case control	Healthy	Both	Adults	Detection of HP IgG ab in the urine	Commercial ELISA	Yes	Not stated	Serum	Various	132	Lower
Osawa et al, 2005; Japan [40]	C	Case control	Sick	Men	Adults	Culture & histology	In-house RIA	Yes	-30	Plasma	Normal	160	Lower
Chuang et al*, 2009; Taiwan [30]	H	Cross-sectional	Sick	Men	Adults	Not described	Commercial RIA	Yes	-72	Plasma	low to normal	145	Lower
Chuang et al*, 2009; Taiwan [30]	H	Cross-sectional	Sick	Women	Adults	Not described	Commercial RIA	Yes	-72	Plasma	low to normal	196	No Difference
Alonso et al, 2007; Spain[42]	P	Cross-sectional	Sick	Both	Adults	UBT, histology (Giemsa stain)	Commercial RIA	Yes	-80	Plasma	Normal	15	Lower
Salles et al, 2006; France[19]	I	Cross-sectional	Sick	Both	Adults	UBT, serology, culture, histology & PCR	Commercial RIA	Yes	-80	Plasma	low to normal	62	Lower
Jun et al, 2007; Korea [37]	J	Cross-sectional	Sick	Both	Adults	RUT, histology (Giemsa stain)	Commercial RIA	Yes	-70	Plasma	Normal	63	No Difference
D'Onghia et al, 2007; Italy [31]	K	Case control	Both	Both	Adults	ELISA	RIA	Yes	-20	Serum	Various	79	Lower
Gao et al, 2009; China [15]	L	Case control	Healthy	Both	Adults	Serology & UBT.	Commercial RIA	Yes	-80	Plasma	Normal	100	Lower
Isomoto et al, 2005; Japan[35]	C	Cross-sectional	Sick	Both	Adults	Anti-IgG antibody, 13C-UBT, or RUT	In-house RIA	Yes	-80	Plasma	Normal	89	Lower
Isomoto et al, 2005; Japan[41]	C	Cross- sectional	Sick	Both	Adults	Serology, UBT or RUT	In-house RIA	Yes	-80	Plasma	Normal	249	Lower
An et al, 2007; Korea[33]	Q	Cohort	Sick	Both	Adults	Not stated	Commercial ELISA	Yes	-70	Plasma	Normal	41	No difference
Nishi et al, 2005; Japan [39]	C	Cross-sectional	Both	Both	Adults	Serology & UBT	In-house RIA	Yes	-80	Plasma	Normal	74	Lower
Czesnikiewicz-Guzik et al, 2005; Poland [12]	B	Cross-sectional	Healthy	Women	Adults	UBT	Not stated	Not stated	Not stated	Serum	Not Stated	100	Lower
Konturek et al, 2006; Poland [17]	B	Cross-sectional	Healthy	Both	Both	UBT & serology	Human RIA	Yes	-80	Serum	Not Stated	180	Lower
Cindoruk et al, 2007; Turkey [11]	M	Cohort	Sick	Both	Adults	Either histology or UBT	RIA	Yes	-80	Plasma	Normal	50	No Difference
Isomoto et al, 2004; Japan [34]	C	Cohort	Sick	Both	Adults	Serology	Commercial RIA	Yes	-80	Plasma	Normal	68	Lower
de Martel, 2007; USA [14]	N	Case control	Sick	Both	Adults	In-house ELISA	Commercial ELISA	Yes	-80	Serum	Various	110	No Difference
Shak et al, 2008, USA[45]	R	Cohort	Healthy	Both	Adults	Serology	Commercial EIA	Yes	-20	Plasma	Obese	24	No difference
Uzzan et al, 2007[44]	S	Cohort	Healthy	Both	Adults	Histology	Commercial RIA	Yes	Not stated	Serum	Obese	13	No difference

* These are from the same paper reporting the same study but were separated because the analysis was stratified by gender and the results for males and females were completely different; hence the decision to separate them.

**Table 6:** Summary of the characteristics of the studies reviewed in Table 5

Characteristic		n	n %
Study Design	Case control	7	25.93
	Cohort	9	33.33
	Cross-sectional	11	40.74
			
Gender studied	Both	21	77.78
	Men	3	11.11
	Women	3	11.11
			
Region	Asia	13	48.15
	Europe	11	40.74
	North America	3	11.11
			
Health status	Both	4	14.81
	Healthy	9	33.33
	Sick	14	51.85
			
Age Group	Adults	23	85.19
	Both	2	7.41
	Children	2	7.41
			
Type of Sickness	Cancer	3	17.65
	GI symptoms	11	64.71
	Others†	3	17.64
			
HP assessment methods used	Not Described	5	18.52
	One	7	25.93
	Two or more	15	55.56
			
Assay Type	Commercial EIA	4	14.81
	Commercial ELISA	3	11.11
	Commercial RIA	14	51.85
	In-house RIA	5	18.52
	Not stated	1	3.7
			
Sample Size	50 and below	6	22.22
	51-200	16	59.26
	201 and above	5	18.52
			
Sample Storage	-70C and above	19	70.37
	Below -70C	4	14.81
	Not Described	4	14.81
			
Sample type	Plasma	17	62.96
	Serum	10	37.04
			
BMI of participants	Normal	15	57.69
	Not Stated	2	7.69
	Obese	2	7.69
	Various	4	15.38
	low to normal	3	11.54

†Others include Crohn's disease (1), diabetes mellitus (1) and multiple conditions (1)

Overall, 17 studies (63%) reported that circulating ghrelin concentrations were lower in *H pylori *positive subjects. Ten studies from Asia (76.9%) and seven from Europe (63.6%) found that circulating ghrelin levels in *H pylori *positive subjects were lower compared to *H pylori *negative subjects. The rest of the studies, including the three from the USA did not find any difference between *H pylori *positive and negative subjects. There was a weak association between the region of the world where the study was conducted and the finding of a lower circulating ghrelin level in *H pylori *positive subjects compared to *H pylori *negative subjects (Fisher's exact test = 0.07). This completely disappeared after controlling for gender, age, health status, type of sample used, storage conditions, BMI, and the sample size.

Zub-Pokrowiecka et al [22] investigated ghrelin changes in the plasma and gastric mucosa among participants with various gastric diseases. Their subjects were divided into four groups - Group 1 had gastric cancer and concomitant *H pylori *infection; group 2 had antral gastritis, duodenal ulcer and *H pylori *infection; group 3 had atrophic gastritis of the fundus and corpus of the stomach but no *H pylori *infection; while group 4 had no gastric lesions and no *H pylori *infection. These researchers reported that the fasting plasma ghrelin concentrations varied among the different groups as follows (in descending order): group 2, group 4, group 1 and group 3.

#### Meta-analysis

**Figure 2:**
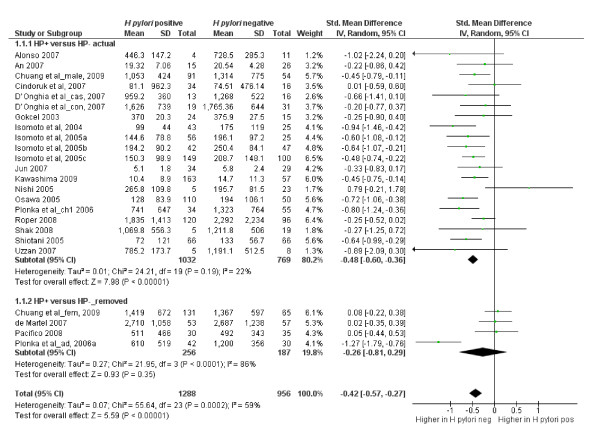
**Forest plot of SMDs of circulating ghrelin concentration between H pylori positive and H pylori negative subjects (the top forest plot labelled 'actual' represents the analysis on which the inference was made**. The lower forest plot labelled 'removed' were studies excluded from the meta-analysis but displayed here to demonstrate their characteristics for readers that might be interested. The values at the bottom of the forest plot represents the overall effect size for both the 'actual' and the 'removed'

**Figure 3:**
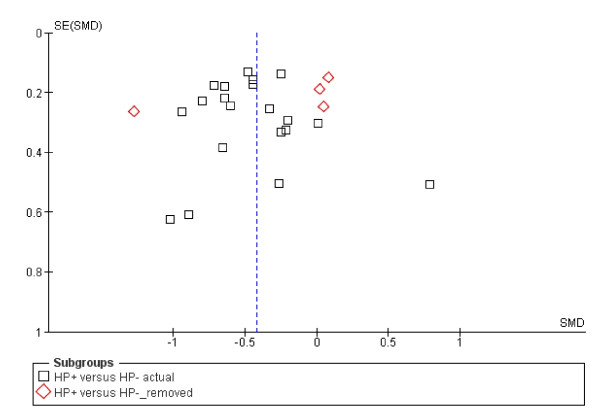
Funnel plot of SMDs of circulating ghrelin concentration between H pylori positive and H pylori negative subjects

Twenty-one of the 26 studies reviewed in this section qualified for inclusion in the meta-analysis. Of these 21 studies, three studies provided ghrelin values for the different categories of subjects studied. Chuang et al [30] presented their results by gender; D'Onghia et al [31] presented their results according to whether the subjects were healthy controls or had colo-rectal cancer; and Plonka et al [32] presented their results for adults and children separately. Because each of these features could affect the circulating ghrelin concentration, the different categories were entered into the meta-analysis as separate papers bringing the total number of papers included to 24 with a total of 2244 participants. Table 4 lists studies excluded from the meta-analysis. Four papers were subsequently removed from the final analysis because they added significant heterogeneity to the analysis (Figure 2). The analysis showed that circulating ghrelin concentration was significantly lower in *H pylori *positive participants than in *H pylori *negative participants (Effect estimate (95%CI) = -0.48 [-0.60, -0.36]). Figure 2 is a forest plot of SMDs of circulating ghrelin concentration between *H pylori *positive and *H pylori *negative subjects. There was no significant heterogeneity among the studies (Heterogeneity: Chi² = 24.21, df = 19 (P = 0.19); I² = 22%). Examination of the funnel plot (Figure 3) suggests some publication bias but the Egger and Begg's tests indicated no publication bias (Begg's test: z = 1.20 (continuity corrected), P = 0.23 (continuity corrected); Egger's bias coefficient = -59.99, P = 0.116). A sensitivity analysis indicated that all the studies included in the meta-analysis contributed approximately equally to the pooled estimate (Table 7). Three of the studies dropped from the meta-analysis did not find any difference in the circulating ghrelin levels between *H pylori *positive and *H pylori *negative subjects [14,26,30]. However, Plonka et al [32] found a significantly lower circulating ghrelin levels among a group of *H pylori *positive Polish shepherds compared to their *H pylori *negative controls. Adding these four papers (excluded because of heterogeneity) only slightly increased the effect estimate (-0.42 [-0.57, -0.27]) but still showed that *H pylori *infection was associated with a lower circulating ghrelin concentration.

**Table 7:** Sensitivity analysis of studies included in the meta-analysis

Studies excluded	SMD (95%CI)	Test for overall effect
Alonso et al, 2007	-0.47 [-0.59, -0.35]	Z = 7.80 (P < 0.00001)
An et al, 2007	-0.49 [-0.61, -0.37]	Z = 7.88 (P < 0.00001)
Chuang et al_male, 2009	-0.48 [-0.61, -0.35]	Z = 7.39 (P < 0.00001)
Cindoruk et al, 2007	-0.49 [-0.61, -0.38]	Z = 8.48 (P < 0.00001)
D'Onghia et al_case, 2007	-0.47 [-0.60, -0.35]	Z = 7.64 (P < 0.00001)
D'Onghia et al_control, 2007	-0.49 [-0.61, -0.37]	Z = 7.95 (P < 0.00001)
Gokcel et al, 2003	-0.49 [-0.61, -0.36]	Z = 7.84 (P < 0.00001)
Isomoto et al, 2004	-0.46 [-0.57, -0.35]	Z = 7.96 (P < 0.00001)
Isomoto et al, 2005a	-0.47 [-0.60, -0.35]	Z = 7.47 (P < 0.00001)
Isomoto et al, 2005b	-0.47 [-0.59, -0.34]	Z = 7.43 (P < 0.00001)
Isomoto et al, 2005c	-0.48 [-0.61, -0.35]	Z = 7.14 (P < 0.00001)
Jun et al, 2007	-0.49 [-0.61, -0.36]	Z = 7.72 (P < 0.00001)
Kawashima et al, 2009	-0.48 [-0.61, -0.35]	Z = 7.32 (P < 0.00001)
Nishi et al, 2005	-0.49 [-0.59, -0.39]	Z = 9.72 (P < 0.00001)
Osawa et al, 2005	-0.46 [-0.58, -0.34]	Z = 7.45 (P < 0.00001)
Plonka et al_ch1 2006	-0.46 [-0.58, -0.34]	Z = 7.65 (P < 0.00001)
Roper et al, 2008	-0.51 [-0.63, -0.39]	Z = 8.37 (P < 0.00001)
Shak et al, 2008	-0.48 [-0.60, -0.36]	Z = 7.79 (P < 0.00001)
Shiotani et al, 2005	-0.46 [-0.59, -0.34]	Z = 7.33 (P < 0.00001)
Uzzan et al, 2007	-0.47 [-0.59, -0.35]	Z = 7.76 (P < 0.00001)

### What is the effect of eradicating H pylori on circulating ghrelin levels?

Table 8 presents an evidence table of the studies that reported the effect of eradicating *H pylori *on circulating ghrelin levels. Thirteen papers were reviewed 12 of which were cohort studies and one RCT. Five of the 13 studies were conducted in Japan; 3 in Korea (both in Asia) and four in Europe. The only study from Africa was an unpublished paper by our team. Table 9 is a summary of the characteristics of the studies in Table 8.

**Table 8:** Evidence table of studies that examined changes in circulating ghrelin levels following *H pylori *eradication

Reference & Country	Study Team	Design	Healthy	Gender	Age category	HP assess	Ghrelin assay Kit	Sample storage	Sample type	Weight	Sample size	Circulating Ghrelin levels after cure	Follow-up (wks)
Nwokolo et al, 2003; UK [2]	A	Cohort	Healthy	Both	Adults	Serology and UBT	Commercial RIA	-20	Plasma	Various	10	Increased	6
Jang et al, 2008; Korea [16]	B	Cohort	Sick	Both	Adults	RUT plus histology & confirmed by UBT	Commercial RIA	-70	Plasma	Normal	16	Increased	Not stated
Osawa et al, 2006; Japan [46]	C	Cohort	Healthy	Men	Adults	Bacterial culture & histology	In-house RIA	Not stated	Plasma	Normal	134	Decreased	12
Czesnikiewicz-Guzik et al, 2007; Poland [13]	D	Cohort	Sick	Women	Adults	UBT; culture of saliva & supragingival dental plaques + serology	Commercial RIA	-80	Plasma	Not stated	49	Increased	4
Lee et al, 2010; Korea [23]	B	RCT	Healthy	Both	Adults	RUT, histology (Giemsa stain)	ELISA	-70	Plasma	Normal	9	No difference	5
Choe et al, 2007, Korea [6]	E	Cohort	Sick	Both	Adults	histology & PCR	Commercial ELISA	-70	Plasma	Normal	8	No difference	4
Pacifico et al, 2008; Italy [26]	F	Cohort	Both	Both	Children	Culture of gastric specimen or histology + RUT	RIA	-70	Serum	Normal	22	Decreased	52
Nweneka, et al, unpublished; Gambia	G	Cohort	Sick	Both	Children	UBT	Commercial RIA	-70	Serum	Low	3	Decreased	4
Isomoto et al, 2005; Japan [36]	C	Cohort	Sick	Both	Adults	RUT, histology (Giemsa stain)	In-house RIA	-80	Plasma	Normal	43	No difference	4
Isomoto et al, 2005; Japan[35]	C	Cohort	Sick	Both	Adults	RUT, histology (Giemsa stain)	In-house RIA	-80	Plasma	Normal	10	No difference	4
Kawashima et al, 2009; Japan [38]	H	Cohort	Both	Both	Adults	Serology	Commercial EIA	-80	Plasma	Normal	49	Increased	23
Cindoruk et al, 2007; Turkey [11]	I	Cohort	Sick	Both	Adults	Either histology or UBT	RIA	-80	Plasma	Normal	23	No difference	12
Isomoto et al, 2004; Japan [34]	C	Cohort	Sick	Both	Adults	Serology	Commercial RIA	-80	Plasma	Normal	9	No difference	4

**Table 9:** Summary of the characteristics of the studies reviewed in Table 8

Characteristic		n	%
Study Design	Cohort	12	92.31
	RCT	1	7.69
			
Gender studied	Both	11	84.62
	Men	1	7.69
	Women	1	7.69
			
Region	Africa	1	7.69
	Asia	8	61.54
	Europe	4	30.77
			
Health status	Both	2	15.38
	Healthy	3	23.08
	Sick	8	61.54
			
Age Group	Adults	11	84.62
	Children	2	15.38
			
Type of Sickness	GI Symptoms	9	90
	PEM	1	10
			
HP assessment methods used	One	3	23.08
	Two or more	10	76.92
			
Assay Type	ELISA	2	15.38
	Commercial RIA	8	61.54
	In-house RIA	3	23.08
			
Sample Size	Above 20	6	46.15
	Below 20	7	53.85
			
Sample Storage	-70°C and below	11	84.62
	Above -70°C	1	7.69
	Not described	1	7.69
			
Sample type	Plasma	11	84.62
	Serum	2	15.38
			
BMI of participants	Normal	10	76.92
	Others	3	23.07
			
Duration of follow-up	4 weeks & below	6	46.15
	Above 4 weeks	6	46.15
	Not recorded	1	7.69
			
Change in ghrelin after cure	Decreased	3	23.08
	Increased	3	23.08
	No difference	7	53.85

In 11 studies, the subjects fasted overnight, and for 3 hours (between 6am and 9am) in one study, before being bled. Osawa et al [46] did not indicate whether their subjects were fasted or not. The sample sizes varied from 3 to 134; median value was 16 (IQR: 9, 43). The duration of follow-up varied from 4 weeks to 52 weeks (median follow-up time: 4.5 weeks; IQR: 4 weeks, 12 weeks). Jang et al [16] were not clear on the duration of follow-up of their subjects.

In seven studies (53.9%) there was no significant difference in circulating ghrelin levels pre- and post *H pylori *eradication. Three studies (23.1%) reported an increase above the pre-eradication level while 3 (23.1%) reported a decrease below the pre-eradication levels. Cross tabulation of the following variables with change in circulating ghrelin levels following cure of *H pylori *infection did not show any association: the age of the participants, the type of sample used, duration of follow-up, sample size, weight of participants, temperature at which the blood samples were stored, the assay kit used to measure circulating ghrelin, the number of methods used to assess *H pylori*, gender, country and the region where the study was conducted. Although not statistically significant, the circulating ghrelin concentration decreased following *H pylori *eradication in the two studies that measured ghrelin using serum samples (Fisher's exact test 0.08). Similarly, the two studies conducted on children found a decrease in circulating ghrelin levels after *H pylori *cure. However, these two studies on children also utilised serum samples; while all the 11 studies conducted in adults used plasma samples. From this descriptive analysis, there is not sufficient data to make a conclusive statement on the effect of *H pylori *eradication on circulating ghrelin levels.

#### Meta-analysis

**Figure 4:**
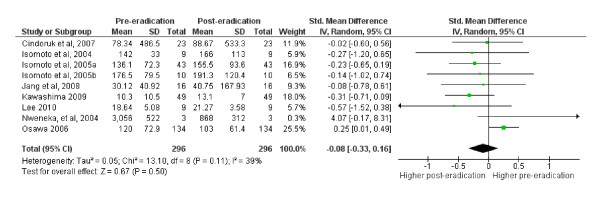
Forest plot of SMDs of circulating ghrelin concentration pre- and post- eradication of h pylori

**Figure 5:**
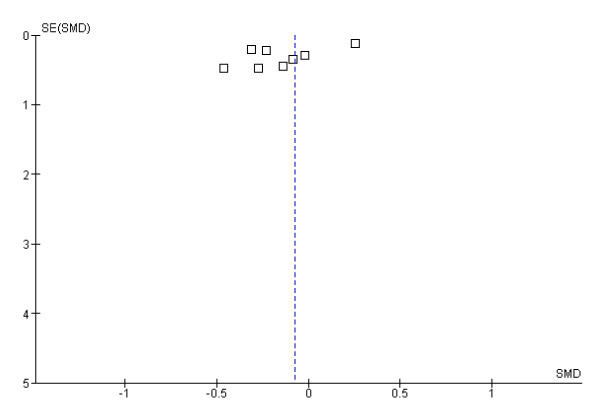
Funnel plot of SMDs of circulating ghrelin concentration pre- and post- eradication of H pylori

Nine (out of the 13 studies reviewed in this section) were included in a meta-analysis with a total population of 592. The excluded studies are listed in Table 4. The analysis showed that eradicating *H pylori *does not have any significant effect on circulating ghrelin levels (Effect estimate (95% CI) = -0.08 [-0.33, 0.16]; Test for overall effect: Z = 0.67 (P = 0.5)). Figure 4 is a forest plot of SMDs of circulating ghrelin concentration pre- and post-eradication of *H pylori*. The funnel plot indicated publication bias (Figure 5).

### What is the effect of H pylori infection on ghrelin expression in the stomach?

Thirteen studies examined the effects of *H pylori *infection on gastric ghrelin. This includes six cohort studies [6,36,44,46-48], 2 case-control studies [40,49], four cross-sectional studies [18,19,24,37] and one randomised controlled trial [23]. The participants included sick subjects (7), well subjects (3) and both well and sick subjects (2). Eight studies were conducted in Asia, two in France, one each in Poland, Brazil and USA. Ten studies recruited both males and females, and three recruited males only. All the sick participants had gastrointestinal symptoms. Different methods were used to assess *H pylori *infection either alone or in combination: histology (11 studies), culture (3), rapid urease assay (5), serology (3), urea breath test (2) and PCR (4). Ten of the 13 studies were conducted on normal weight subjects, two on obese subjects and one on subjects of different body weights. Due to the different gastric ghrelin parameters assessed, a meta-analysis was not possible.

Five studies assessed gastric ghrelin contents: one found it to be lower in *H pylori *positive subjects [36], three [6,18,44] found no significant difference between *H pylori *positive and negative participants, and one [24] found increased levels in *H pylori *positive participants. Isomoto et al [36] compared gastric ghrelin peptide contents in the endoscopic biopsies from the corpus of 56 *H pylori *positive and 25 *H pylori *negative subjects using radio-immunoassay. They reported significantly lower gastric ghrelin content in the *H pylori *positive subjects than the *H pylori *negative subjects. Roper et al [18] studied 216 adult males of normal BMI presenting for routine endoscopy consisting of 120 *H pylori *positive and 96 *H pylori *negative subjects. Although they did not find any significant difference in the gastric ghrelin levels between *H pylori *positive and *H pylori *negative subjects, they reported a very wide variation in the concentration of ghrelin in the gastric juice (from <80 to 776,000 pg/ml) with the *H pylori *positive subjects having higher gastric juice ghrelin levels than the *H pylori *negative subjects. Choe et al [6] did not find any significant difference in the gastric ghrelin levels between *H pylori *positive and *H pylori *negative subjects using biopsied tissues.

Four studies examined the expression of ghrelin mRNA in gastric mucosa [19,36,37,40]. Three studies found ghrelin mRNA expression to be lower in *H pylori *positive subjects than in *H pylori *negative subjects, while Jun et al [37] found no difference. Five studies assessed the quantity of ghrelin immunoreactive cells in the gastric mucosa [36,40,47-49]; all of which found that *H pylori *positive subjects had fewer ghrelin-producing cells than in uninfected subjects. However, in the study by Isomoto et al [36], this difference did not achieve statistical significance.

Five studies compared the various gastric ghrelin parameters before and after *H pylori *eradication [6,23,36,46,48]. Choe et al [6] did not find any significant difference in the ghrelin concentration in the antrum, corpus and fundus pre- and post- *H pylori *eradication. Lee et al [23] reported a randomized controlled trial on *H pylori *positive volunteers without peptic ulcer or any other gastrointestinal symptoms in which the treatment group received triple *H pylori *eradication regimen while the control group did not receive any treatment. These authors reported a significant increase in gastric ghrelin mRNA expression following eradication compared to the control group. Osawa et al [46] also reported an increase in ghrelin mRNA expression following *H pylori *cure. Although Isomoto et al [36] found a tendency towards increase in ghrelin mRNA expression following cure of *H pylori*, this increase was not significant. Tatsuguchi et al [48], on the other hand, found an increase in ghrelin immunoreactive cells following *H pylori *eradication in 50 patients with either peptic ulcer disease or gastritis, while in 11 patients who did not respond to eradication therapy, there was no difference in the number of ghrelin immunoreactive cells pre-and post-eradication therapy.

Three studies [36,47,49] found a negative correlation between ghrelin producing cells in the gastrum and the severity of *H pylori *infection. Gastric ghrelin content and gastric ghrelin mRNA expression were both positively correlated with plasma ghrelin concentration [36,46].

## Discussion

The potential role of ghrelin in body mass regulation makes understanding its interactions with *Helicobacter pylori*, a highly prevalent gastrointestinal infection, important. To address this issue, we asked three questions: how does *H pylori *infection affect circulating ghrelin levels; how does eradicating *H pylori *affect circulating ghrelin; and how does *H pylori *infection affect gastric ghrelin and ghrelin producing cells.

The results of our analysis has conclusively shown that circulating ghrelin levels are significantly higher in *H pylori *negative people than in those infected with *H pylori *(P = 0.00001). Our results also suggest that eradicating *H pylori *does not have any significant effect on circulating ghrelin. Although there was no significant heterogeneity between the group of studies that compared circulating ghrelin concentrations before and after *H pylori *eradication, this result should be interpreted with the following caveats in mind: 1) three of the studies [34-36] included in the meta-analysis came from the same research group; and together accounted for 29% of the effect size; 2) the paper by Osawa et al [46] which reported higher ghrelin levels before *H pylori *eradication compared to the levels after eradication on its own contributed 26.2% of the effect; and 3) the sample sizes of most of the other studies were relatively small. Interestingly, all the smaller studies, except one, found higher circulating ghrelin levels post-eradication. Even among the subjects studied by Osawa et al [46], plasma ghrelin increased in 50 patients and decreased in 84 patients, although the overall effect was a decrease post-eradication. They were however, able to show that pre-eradication elevation of ghrelin was associated with a fall in ghrelin post-eradication. In our unpublished data, we also found that elevated ghrelin concentration pre-eradication was associated with a fall post-eradication. These two observations suggest that in addition to other factors, the pre-eradication ghrelin level determines the direction of response of ghrelin post-eradication.

The heterogeneity of the studies examining the effect of *H pylori *on gastric ghrelin expression as well as the small number of studies that examined different aspects of this relationship did not allow a meta-analysis to be performed. However, the descriptive data suggests that available evidence is still discrepant; although the weight of evidence seems to favour lower ghrelin mRNA and ghrelin immunoreactive cells in association with *H pylori *infection [19,36,40,47-49]. The ultimate effect of *H pylori *on gastric ghrelin appears to be dependent on the duration of infection and the extent of *H pylori*-induced damage to the gastric mucosa. At least three studies found a negative correlation between number of ghrelin producing cells and the severity of *H pylori *infection [36,47,49].

The close anatomical proximity between the site of *H pylori *infection and the site of ghrelin production might result in the loss of ghrelin producing cells as part of the *H pylori *associated gastritis, causing reduced ghrelin production. However, such effect will not be restricted to *H pylori *infection and could occur in any other condition associated with gastritis. For example, Checchi et al [10] studied 233 patients with autoimmune gastritis (indicated by elevated parietal cell antibody (PCA)) and 211 control subjects, and found that mean serum ghrelin levels in PCA positive patients were significantly lower than in PCA negative patients, similar to the results found in *H pylori *infection by some studies. This decrease remained significant even after excluding patients with *H pylori *infection, suggesting that the *H pylori *infection was not necessarily responsible for the reduction in serum ghrelin. Again, the region of the stomach biopsied could also affect the results. Jang et al [16] reported that after ulcer healing and *H pylori *eradication, there was a significant increase in the levels of ghrelin mRNA. But while corpus ghrelin mRNA increased after cure and *H pylori *eradication, anthral ghrelin mRNA decreased, suggesting a differential response by the ghrelin producing cells in the different regions of the stomach. In healthy *H pylori *infected subjects however, Lee et al [23] found a significant increase in fundic ghrelin mRNA after eradication of *H pylori *(P = 0.0002) but no change in the anthral ghrelin mRNA (P = 0.5), suggesting a more complex relationship between gastric ghrelin production and *H pylori *infection. Teasing out this relationship will require more rigorous investigation.

If *H pylori *infection is associated with a lower circulating ghrelin, it is biologically plausible that its eradication will be associated with an increase in circulating ghrelin. But if *H pylori *reduces circulating ghrelin by destroying ghrelin producing cells, then the effect of eradicating it on circulating ghrelin would depend on the duration of infection, the amount of damage to ghrelin-producing cells, and the time it takes for these cells to regenerate. Indeed several studies have found a negative correlation between the number of ghrelin producing cells and the severity of gastritis [36,47,49]. And in one study, there was no change in ghrelin levels post-eradication after 4 weeks of follow-up, but the level progressively increased with follow-up, achieving, in some subjects, significant increase after 6 months of follow-up [36]. In contrast, mice infected with *H pylori *for 6-8 months had higher ghrelin levels compared to time-matched controls, which normalised two months post-eradication [50]. However, Masaoka et al [51] did not find any change in circulating ghrelin levels 2 years after successful eradication of *H pylori *in an adult man.

The discrepancy in the response to *H pylori *eradication could also be related to the strain of *H pylori*. Isomoto et al [52] found that strain diversity in *H pylori *was associated with plasma and gastric ghrelin levels in humans. Patients with type I strain (which express the virulence factors cytotoxin-associated gene product (CagA) and Vacuolating cytotoxin A (VacA)) have lower circulating ghrelin levels than those with the less virulent type II strain which does not express the virulence factors. This finding also argues for a possible racial difference in the association between circulating ghrelin levels and *H pylori *infection: in regions where type I strain is dominant, one would expect to see reduced circulating ghrelin levels. While such speculation is attractive, it might not be entirely correct [53]. In this review, the effect of region and country of study on the relationship between *H pylori *and ghrelin was very weak, and appears to be confounded by several other factors.

The underlying clinical condition of the subject might also be affecting the results. Suzuki et al [54] studied plasma ghrelin in patients with peptic ulcer disease. They found that plasma ghrelin levels were significantly higher in patients with duodenal ulcer as well as those with gastric ulcer compared to those with chronic gastritis. Among the subjects that were *H pylori *positive, plasma ghrelin was significantly higher in patients with duodenal or gastric ulcer than in those with non-ulcer chronic gastritis. After treatment for the ulcer (with healing), no significant change was found in the plasma ghrelin levels (i.e. pre-and post eradication levels were similar). Most of the studies reviewed here recruited subjects with varying degrees and types of gastrointestinal pathology. If each of these gastrointestinal diseases affects ghrelin production differently as suggested by Suzuki et al [54], then the discrepancies noted in this review are to be expected. Compensation from other sources of ghrelin production might also explain the various inconsistencies highlighted in this review. For example, Suzuki et al [55] infected Mongolian gerbils with *H pylori *and assessed the plasma and gastric ghrelin levels at 17 and 23 weeks after the infection. They found a significant decrease in gastric ghrelin in the *H pylori *positive gerbils compared to the controls, but also found a significant increase in plasma ghrelin levels in the *H pylori *positive group. This same group also found increased plasma ghrelin and decreased gastric ghrelin levels in IL-1R1 knockout mice [4] suggesting that other sources of ghrelin might have contributed to the increased plasma ghrelin.

### Limitations and implications for research and practice

The major limitation of this review is the use of only English language papers, which raises the possibility of some publication bias. Another limitation in conducting this review is that all except one of the papers were observational studies, most of which did not primarily set out to assess the relationship between ghrelin and H pylori; but assessed both parameters in relation to other objectives. Well designed randomised clinical trials are needed to verify the conclusions made by this review. Also, many of the studies included participants with diverse disease conditions whose impacts on ghrelin secretion have not been investigated before. The best approach to solve the riddle of the relationship between ghrelin and helicobacter pylori might be to study otherwise healthy participants with asymptomatic *H pylori *infection.

## Conclusions

From available evidence, circulating ghrelin concentration is lower in people infected with *H pylori *compared to those not infected with the bacterium. However, a more complex relationship exists between circulating ghrelin levels and eradication of *H pylori*. This relationship may be modulated by the strain of infecting *H pylori*, the duration of follow-up, the extent of *H pylori*-induced gastritis and other underlying pathology. More studies are needed to further elucidate the impact of *H pylori *eradication on circulating ghrelin concentration.

## Competing interests

The authors declare that they have no competing interests. Funding for this study was provided by the UK Medical Research Council.

## Authors' contributions

CVN conceived the paper, conducted the literature search, initial analysis and took lead in the writing. AMP critically reviewed the analysis, and the initial drafts, and participated in writing subsequent drafts. Both authors read and approved the final manuscript.

## Pre-publication history

The pre-publication history for this paper can be accessed here:

http://www.biomedcentral.com/1471-230X/11/7/prepub
